# Boosting immune response with the invariant chain segments via association with non-peptide binding region of major histocompatibility complex class II molecules

**DOI:** 10.1186/1471-2172-13-55

**Published:** 2012-09-27

**Authors:** Fangfang Chen, Fantao Meng, Ling Pan, Fazhi Xu, Xuelan Liu, Weiyi Yu

**Affiliations:** 1 Key Laboratory of Zoonoses of Anhui Province, Anhui Agricultural University, 130 Changjiang West Road, Hefei 230036, China

**Keywords:** Li-segments, Epitope, Hybrid, MHC II, Antibody, Membrane co-localization

## Abstract

**Background:**

Based on binding of invariant chain (Ii) to major histocompatibility complex (MHC) class II molecules to form complexes, Ii-segment hybrids, Ii-key structure linking an epitope, or Ii class II-associated invariant chain peptide (CLIP) replaced with an epitope were used to increase immune response. It is currently unknown whether the Ii-segment cytosolic and transmembrane domains bind to the MHC non-peptide binding region (PBR) and consequently influence immune response. To investigate the potential role of Ii-segments in the immune response via MHC II/peptide complexes, a few hybrids containing Ii-segments and a multiepitope (F306) from Newcastle disease virus fusion protein (F) were constructed, and their binding effects on MHC II molecules and specific antibody production were compared using confocal microscopy, immunoprecipitation, western blotting and animal experiments.

**Results:**

One of the Ii-segment/F306 hybrids, containing ND (Asn–Asp) outside the F306 in the Ii-key structure (Ii-key/F306/ND), neither co-localized with MHC II molecules on plasma membrane nor bound to MHC II molecules to form complexes. However, stimulation of mice with the structure produced 4-fold higher antibody titers compared with F306 alone. The two other Ii-segment/F306 hybrids, in which the transmembrane and cytosolic domains of Ii were linked to this structure (Cyt/TM/Ii-key/F306/ND), partially co-localized on plasma membrane with MHC class II molecules and weakly bound MHC II molecules to form complexes. They induced mice to produce approximately 9-fold higher antibody titers compared with F306 alone. Furthermore, an Ii/F306 hybrid (F306 substituting CLIP) co-localized well with MHC II molecules on the membrane to form complexes, although it increased antibody titer about 3-fold relative to F306 alone.

**Conclusions:**

These results suggest that Ii-segments improve specific immune response by binding to the non-PBR on MHC class II molecules and enabling membrane co-localization with MHC II molecules, resulting in the formation of relatively stable MHC II/peptide complexes on the plasma membrane, and signal transduction.

## Background

Major histocompatibility complex (MHC) class II molecules play an important role in antigen presentation, with the binding antigen peptide as a key step in initiating the specific immune response. In this process, the invariant chain (Ii) acts as a chaperone for the correct folding and the functional stability of MHC II molecules [1]. Ii is described initially as a nonpolymorphic type II integral membrane protein [2], binding MHC II α and β chains to form αβ/Ii complexes. These αβ/Ii complexes have been identified on B cell and dendritic cell surfaces [3-5]. In epithelial cells, the endocytosis of αβ/Ii complexes is highly dependent on the Ii di-leucine motif [6,7]. Ii limits HLA-DR egress from the endoplasmic reticulum [8] and prevents loading of self-peptide [2,9,10]. In immature lysosomes, Ii is proteolyzed [11], and its class II-associated invariant chain peptide (CLIP) is replaced by an antigenic peptide [12]. In B cells, the major pathway of Ii-associated MHC class II molecules involves direct access to the endolysosomal compartments for peptide loading [13].

The mouse Ii isoform Ii31 consists of the cytosolic and transmembrane domains and luminal domain that contains CLIP and the trimerization region [14]. The cytosolic domain (Cyt) contains an endosome-targeting signal that is essential for Ii targeting to the endosomal compartment, via the plasma membrane alone or the MHC class II complex [15,16]. The transmembrane domain (TM) plays a key role in the formation of Ii trimers [17] and in the degradation of Ii [18], thereby influencing MHC class II molecular functions, including complex formation and antigen presentation [19]. The CLIP binds MHC class II peptide binding region (PBR) and interacts with class II molecules [10,20]. The trimerization region is involved in the generation of this endosomal localization signal [17].

A method to increase antigen-specific stimulation of T-helper cells entails the use of the Ii hybrids, in which a four-amino-acid sequence (LRMK) is linked to T-helper epitopes [21-23], or the Ii peptide (CLIP) region is replaced with the various epitopes [24,25]. Animal models illustrate the efficiency of Ii hybrid methodology in using melanoma peptides [21,26], subvirion influenza A (H5N1) HA [22], human papilloma virus 16 E7(8–22) [23], *Listeria* Th [24] and hepatitis C virus [25] epitopes. The mechanistic hypothesis states that the Ii -key binds initially to an allosteric site just outside the MHC class II binding groove at the cell surface [26,27]. This induces a conformational change in the trough, facilitating antigenic epitope charging [22,28], and a concomitant increase in the potency of antigen presentation compared with the unmodified class II epitope [29,30]. As vector, Ii-key and Ii can enhance the interferon (IFN)γ and interleukin (IL)-4 or IL-2 responses in enzyme-linked immunosorbent spot assay [21,24], epitope-specific CD4^+^ T cell activation [23], or specific antibody production [25]. The Ii-hybrids can also function in desensitizing allergy [31] and inducing antigen-specific tolerance and ameliorating arthritis [32]. All these findings indicate potential clinical use of such allosteric site-directed, Ii-segment drugs [27].

**Figure 1:**
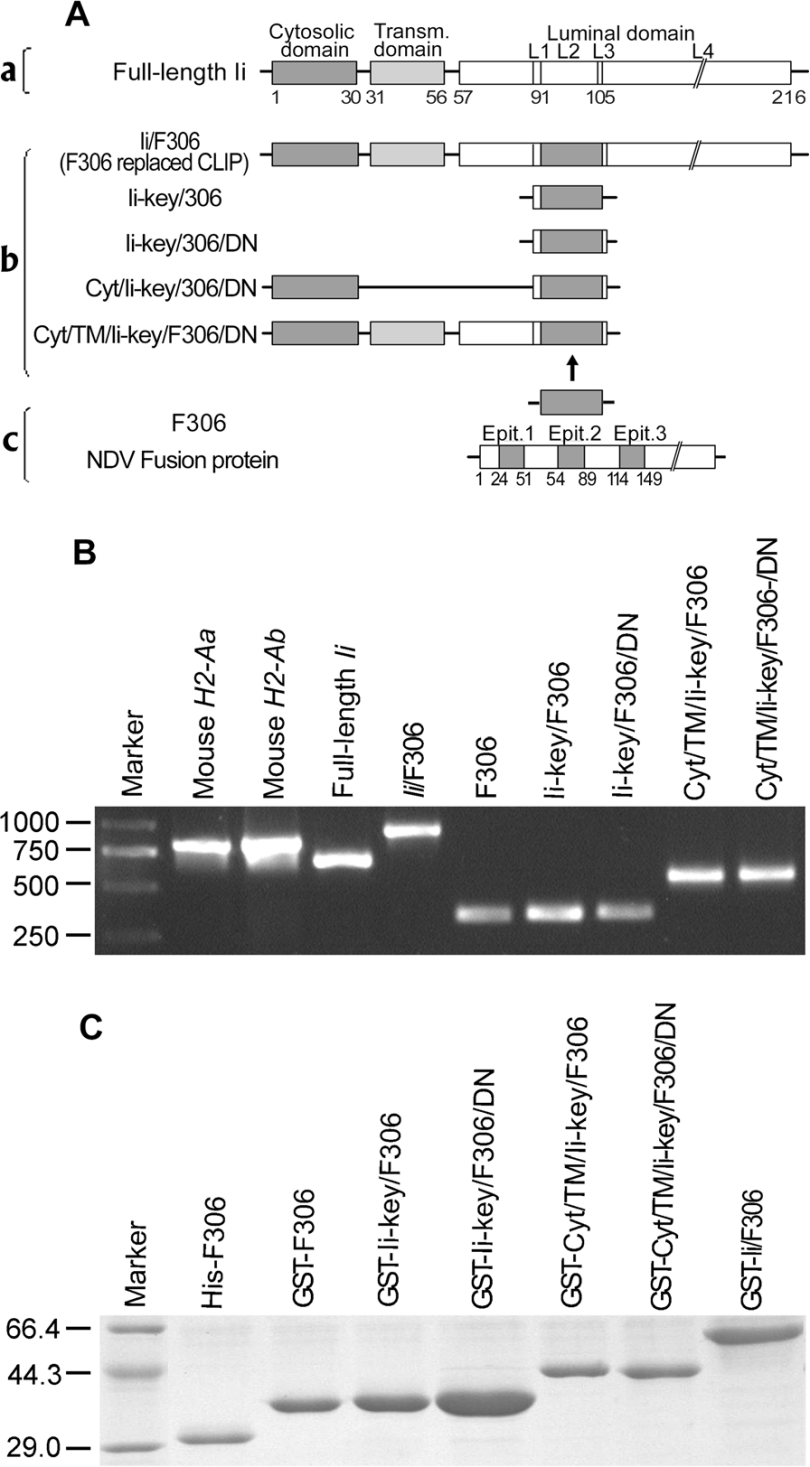
**Constructed, expressed and purified hybrids containing Ii-segments and antigen peptides. A**. Schematic diagram of hybrids containing Ii-segments and antigen peptides. **a**. Full-length Ii consists of cytosolic domain (Cyt), transmembrane domain (TM) and luminal domain, which includes an Ii-key sequence **(**L1**)**, CLIP **(**L2**)**, DN sequence **(**L3**)** and trimerization region **(**L4**)**. **b**. Components of reconstructed hybrids. These hybrids contain different Ii-segments and a multiepitope, F306. **c**. Structure of F306. F306 consisted of three potential epitopes in fusion protein of Newcastle disease virus and had a predicted molecular weight of 11.2 kDa. **B**. PCR-amplified gene and hybrid DNA segment products: mouse *H2-Aa *, *H2-Ab *, full-length *Ii *, *Ii */F306, multi-epitope F306 and the hybrids, Ii-key/F306, Ii-key/F306/DN, Cyt/TM/Ii-Key/F306/ and Cyt/TM/Ii-Key/F306/DN. **C**. Expressed and purified products. PCR-amplified F306 and hybrid DNA were cloned into prokaryotic expression vectors, pGEX-4 T-1 or pET-32a, and expressed. Purified products: GST-Ii/F306, GST-F306, GST-Ii-key/F306, GST-Ii-key/F306/DN, GST-Cyt/TM/Ii-Key/F306, and GST-Cyt/TM/Ii-Key/F306/DN for immunization and His-F306 for coating in ELISA.

The Ii-key lies outside the N-terminal of CLIP and plays an important role in CLIP loading in the MHC II PBR. Hypothetically, the DN (Asn–Asp) segment, lying just outside the C-terminal of CLIP (Figure 1A), would promote epitope association with the PBR, in a similar manner to the Ii-key. Furthermore, some of the Ii-segments have a potential immune function [19,20,29]. The cytosolic and transmembrane domains are involved in localization and binding to MHC class II molecules, with the former containing an endosome-targeting signal [15,16]. It is unclear whether the Ii-segments in the hybrids are able to induce a conformational change that enables antigenic peptide charging, stabilizing the MHC II/peptide complexes and enhancing specific immune responses, when Ii hybrid binds MHC II molecules to form complexes, in which the epitope binds to the PBR and the other segments to the non-PBR. Therefore, we constructed such hybrids to determine their binding effect with MHC II molecules, and antibody production.

## Results

### Construction and identification of the Ii-segment epitope hybrids

We amplified Ii-segments from the mouse Ii cDNA generated in our previous work [33], and then constructed the hybrids containing fusion genes of Ii-segments and a multiepitope containing three potential epitopes, named F306 [34], from Newcastle disease virus fusion protein (F). A schematic diagram in Figure 1A shows the structures of these hybrids. After identification with electrophoresis and sequencing, they (*Ii*, *Ii*/F306, F306, Ii-key/F306, Ii-key/F306/DN, Cyt/Ii-Key/F306/, Cyt/TM/Ii-Key/F306/DN) (Figure 1B) were inserted into the eukaryotic expression vectors pmCherry-C1 for transfection or the prokaryotic expression vectors to construct pGEX-4 T-1 for expression in *Escherichia coli* for immunization (GST- Ii/F306, GST-F306, GST-Ii-key/F306, GST-Ii-key/F306/DN, GST-Cyt/TM/Ii-Key/F306, and GST-Cyt/TM/Ii-Key/F306/DN). pET-32a-F306 was constructed in order to purify F306 multiepitope which in turn was used as a coating antigen for ELISA. These expressed proteins were purified and identified (Figure 1C). Additionally, mouse *H2-Aa*, *H2-Ab* and full-length *Ii* genes (Figure 1B) were also amplified and inserted in eukaryotic expression vectors (Table 1) for transfection, immunoprecipitation or western blotting, respectively. 

**Table 1:** Primers, cloned Ii-segments and reconstructed vectors in this study

**No.**		**Primer sequences (5´-´)**	**Reconstructed vectors**
1	Forward:	5'gaagatctaatgccgtgcagcagagc3'	pEGFP-N1-*H2-Aa* (1)
	Reverse:	5'gcgtcgacactaaaggccctggg3'	
2	Forward:	5'gaagatcttatggctctgcagatcccc3'	pEGFP-N1-*H2-Ab* (1)
	Reverse:	5'gcgtcgacatcgcaggagccct3'	
3	Forward:	5'cccgaattcacatggctctgcagatccc3'	pCMV-Myc-*H2-Ab* (2)
	Reverse:	5'ggaagatctatcactgcaggagccct3'	
4	Forward:	5'cccgaattctatggctctgcagatcccc3'	pmCherry-C1-*Ii* (1)
	Reverse:	5'gcgtcgactcacagggtgacttga3'	pEGFP-C1-*Ii* (3)
5	Forward1:	5'ccgctcgagacatgcaacgcgacct3'	pmCherry-C1-Ii/F306 (1)
	Reverse1:	5'atgagcaagaactccctggaggagaag	pEGFP-C1-Ii/F306 (3)
	Forward2:	aagcccacagagg3'	
	Reverse2:	5'ctcctccagggagttcttgctcatctcaaa	
		caagagccactgc3'	
		5'cgggatcctcacagggtgacttgaccc3'	
6	Forward:	5'gaattcgatgcttcgcatgaagcttcc3'	pmCherry-C1-Ii-Key/CLIP/DN (1)
	Reverse:	5'gtcgacctagttatccatgaacat3'	
7	Forward:	5'ccggaattcgatgcaacgc3'	pmCherry-C1-Cyt/Ii-Key/CLIP/DN (1)
	Reverse:	5'gtcgacctagttatccatggacat3'	
8	Forward:	5'gaattcgatgcttcgcatgaagcttcc3'	pmCherry-C1-Cyt/TM/Ii-Key/CLIP/DN (1)
	Reverse:	5'gtcgacctagttatccatggacat3'	
9	Forward:	5'ccgctcgagacatgcttcgcatgaag3'	pmCherry-C1-Ii-key/F306/DN (1)
	Reverse:	5'cgggatcctcagttatcataaatacc3'	pEGFP-C1-Ii-key/F306/DN 3)
10	Forward:	5'ccggaattcgatgcaacgc3'	pmCherry-C1-Cyt/Ii-Key/F306/DN (1)
	Reverse:	5'ccgctcgagctagttatccat3'	pEGFP-C1-Cyt/Ii-Key/F306/DN 3)
11	Forward:	5'ccgctcgagatatgcaacgcgacctca3'	pmCherry-C1-Cyt/TM/Ii-Key/F306/DN (1)
	Reverse:	5'cggcatcctcagttatcataaatacc3'	
12	Forward:	5'ccggaattcatgctcccaaatatg3'	pEGFP-C1-Cyt/TM/Ii-Key/F306/DN (3)
	Reverse:	5'ccgctcgagtcaataaataccagg3'	
13	Forward:	5'ccggaattcatgcttcgcatgaagctc3'	PGEX-4T-1-Ii-key/F306 (4)
	Reverse:	5'ccgctcgagtcaataaataccaggag3'	
14	Forward:	5'ccggaattcatgcttcgcatgaagctc3'	PGEX-4T-1-Ii-key/F306/DN (4)
	Reverse:	5'ccgctcgagtcaataaataccagg3'	
15	Forward:	5'cgggatccatgcaacgcgacct3'	PGEX-4T-1-Cyt/TM/Ii-Key/F306 (4)
	Reverse:	5'ccgctcgagtcaataaataccag3'	
16	Forward:	5'cgggatccatgcaacgcgacct3'	PGEX-4T-1-Cyt/TM/Ii-Key/F306/DN (4)
	Reverse	5'ccgctcgagtcagttatcaccagga3'	
17	Forward:	5'cgggatccatgcaacgcgacct3'	PGEX-4T-1-Ii/F306 (4)
	Reverse:	5'cgggatcctcacagggtgacttgaccc3'	
18	Forward:	5'ccggaattcatgctcccaaatatgcctaag3'	PGEX-4T-1-F306 (4)
	Reverse:	5'ccgctcgagtcaataaataccaggagacataggg3'	pET-32a-F306 (5)

The design of all the primers was based on the reported cDNA sequences: mouse *Ii *from GenBank ID: NM_010545, mouse *H2-Aa *from GenBank ID: NM_010378, mouse *H2-Ab *from GenBank ID: AY_452202 and NDV *F *gene from GenBank ID: AY_508514. The reconstructed vectors were used for CLSM (1), for IP (2), for WB (3), for expression as immunization antigens (4)or for expression as coating antigen in ELISA (5).

### Ii-segments in hybrids bound to non-PBR of MHC class II molecules

**Figure 2:**
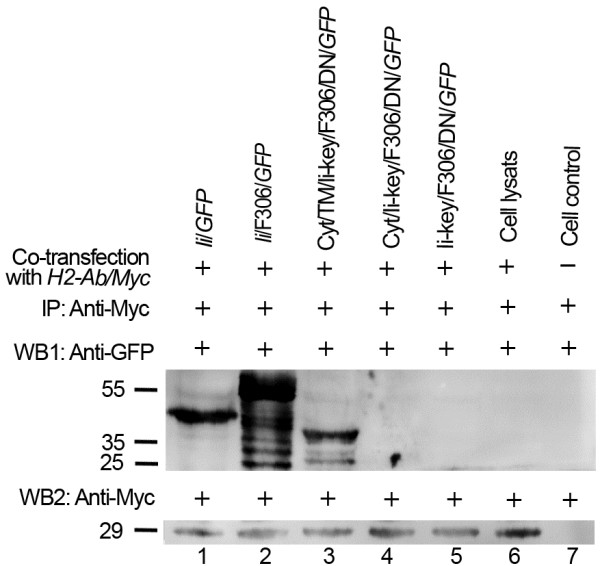
**Binding of Ii, Ii-segments, Ii-segments/F306 or Ii/F306 hybrids to MHC II β chain. **Mouse *H2-Ab/Myc *was cotransfected with mouse *Ii *gene *Ii/GFP *(1), *Ii */F306 hybrids (2), Cyt/TM/Ii-key/F306/DN/*GFP *(3), Cyt/Ii-key/F306/DN/*GFP *(4) or Ii-key/F306/DN/*GFP *(5) in COS7 cells. After 36 h, the cells were lysed and the antibody against Myc was added for immunoprecipitation. Subsequently, the immune complexes (lanes 1–5), cell lysates transfected with only *H2-Ab/Myc *(lane 6) and control cells without transfection (lane 7) were separated by SDS-PAGE, immunoblotted and detected with antibodies against GFP (WB1) or Myc (WB2). These fusion proteins had molecular weights of 51.0 kDa (Ii/GFP), 58.9 kDa (Ii/F306/GFP), 45.7 kDa (Cyt/TM/Ii-key/F306/DN/GFP), 31 kDa (Cyt/Ii-key/F306/DN/GFP) and 27.7 kDa (Ii-key/F306/DN/GFP), in which GFP had a molecular weight of 27 kDa.

Complexes involving MHC II α, β and Ii (αβ/Ii) at the cell surface [3-7] resulted from binding with hybrids containing different Ii-segments. We carried out immunoprecipitation and western blotting to establish how Ii segments in the hybrids bound to MHC class II molecules. The results indicated that the full-length Ii and the Ii/F306 hybrid bound MHC II β chain strongly (Figure 2, lanes 1 and 2) (α chain data not presented). Another hybrid containing the transmembrane and cytosolic domains (Cyt/TM/Ii-key/F306/DN) also bound MHC class II molecules (Figure 2, lane 3), and the hybrids containing cytosolic domain (Cyt/Ii-key/F306/DN) or containing only Ii-key and DN (Ii-key/F306/DN) (Figure 2, lanes 4 and 5) failed to bind MHC class II molecules. These results indicated that besides the trimerization region, the other segments, such as the cytosolic and transmembrane domains together (Cyt/TM), had a role in binding to MHC class II molecules. Because Ii or Ii hybrids bind to the MHC II molecule, Ii CLIP region or the epitope F306 in the Ii hybrids loaded in the PBR, Ii functional segments might bind non-PBRs of MHC II molecules. 

### Some Ii segments co-localized with MHC class II molecules on the plasma membrane

**Figure 3:**
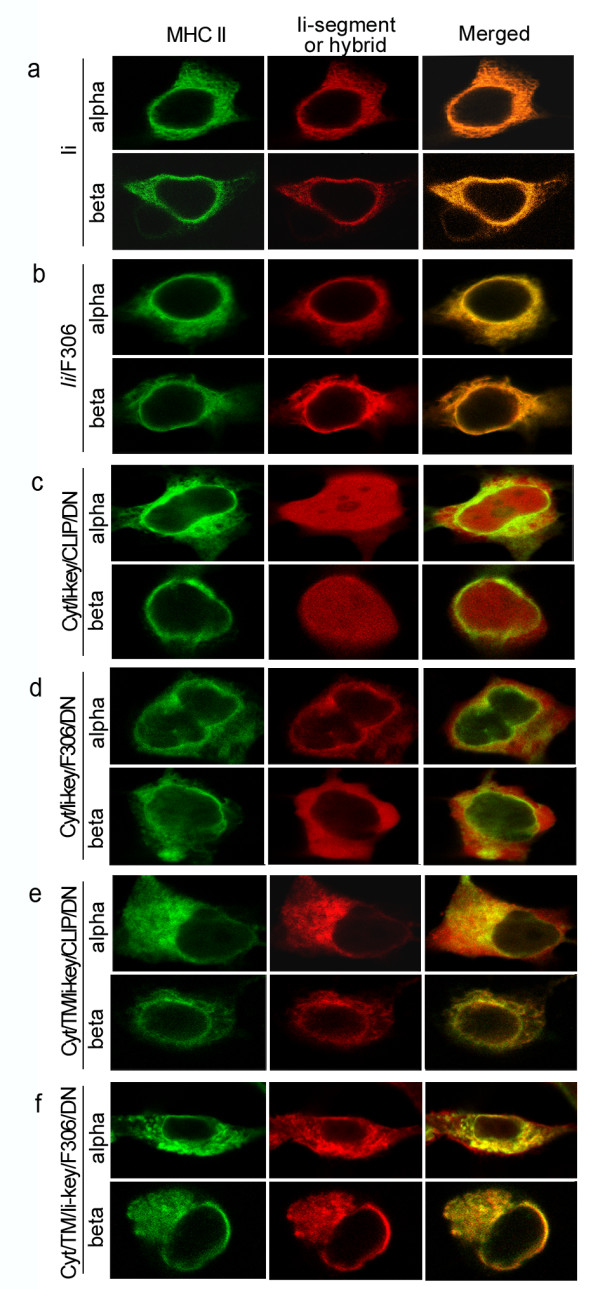
**Interaction between MHC class II molecule and Ii-segments or hybrids in co-transfected COS7 cells. **Cells were transiently co-transfected with the reconstructed pEGFP-N1-*H2-Aa * or pEGFP-N1-*H2-Ab *expressing gene of fusion protein (GFP/MHC class II alpha or beta chain) and the reconstructed pmCherry-C1s expressing genes of fusion protein (RFP/Ii hybrids or RFP/Ii-segments), respectively. After 24 h, the cells were observed using CLSM and × 60 oil objective. a) Mouse Ii and MHC II alpha or beta chain. b) Mouse MHC II alpha or beta chain and Ii/F306 hybrid. Both molecules co-localized with MHC II molecules to plasma membrane visualized in the merged images represented in orange. c) Mouse MHC II alpha or beta chain and Cyt/Ii-key/CLIP/DN. d) Mouse MHC II alpha or beta chain and Cyt/Ii-key/F306/DN. Both Ii-segments localized in cytosol or to plasma membrane and appeared with absent co-localization with MHC II molecules as visualized in the merged image. e) Mouse MHC II alpha or beta chain and Cyt/TM/Ii-key/CLIP/DN hybrids. f) Mouse MHC II alpha or beta chain and hybrids Cyt/TM/Ii-key/F306/DN. Both hybrids localized on plasma membrane, but appeared with relative weak interaction with MHC II molecules visualized in the merged image represented in dispersive yellow-orange, respectively.

To determine where in the cells the segments bind MHC class II α or β chains, we cotransfected COS7 cells with these segments or hybrids as well as *H2-Aa* or *H2-Ab*. MHC II α or β chains (Figure 3a–f, left), full-length Ii (Figure 3a, middle) and Cyt/TM/Ii-key/CLIP(F306)/ND (Figure 3d and f, middle) were expressed on the plasma membrane in the transfected COS7 cells, but the Ii-segments, Cyt/Ii-key/CLIP/DN (Figure 3c middle) were not expressed. The complete co-localization of full-length Ii and Ii/F306 hybrid with MHC II α or β chain on the plasma membrane resulted in the appearance of a uniform range or yellow-orange accumulation in the merged images (Figure 3a and b, merged). It depended on molecular integrity, because full-length Ii and Ii/F306 hybrid contained the cytosolic and transmembrane domains for membrane localization and the trimerization region for polymerization. In the presence of cytosolic and transmembrane domains, however, the Ii-segments (Cyt/TM/Ii-key/CLIP/ND) and Ii-segment/epitope hybrid (Cyt/TM/Ii-key/F306/ND) resulted in partial co-localization with MHC II α or β chain, which was consistent with the imaging results observed in Figure 3d and f. All the other Ii-segments and hybrids, which lacked the transmembrane domain and trimerization region, such as Cyt/Ii-key/CLIP/ND, Ii-key, Ii-key/CLIP, Ii-key/CLIP/DN (images not presented) and Cyt/Ii-key/F306/ND, did not bind to MHC II molecules (Figure 2, lanes 4 and 5), even if they were localized in the cytosol (Cyt/Ii-key/CLIP/ND) (Figure 3c, middle) or cell membrane (Cyt/Ii-key/F306/ND) (Figure 3e, middle). The differences in the merged images (Figure 3a, b, d and f, merged) hypothetically resulted from the degree of tight binding of the hybrids with MHC class II molecules.

### All the Ii-segments increased immune response

**Figure 4:**
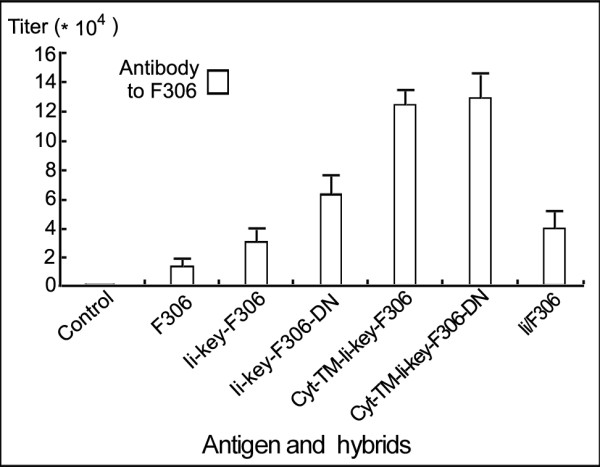
**Effect of the Ii-segments on F306-specific antibody responses in the different hybrid immunized Balb/c mice. **Balb/c mice were injected i.p with antigen: F306, Ii hybrids (GST-Ii-key/F306, GST-Ii-key/F306/DN, GST-Cyt/TM/Ii-key/F306, GST-Cyt/TM/Ii-key/F306/DN or GST-Ii/F306) at week 0, and boosted at weeks 2 and 3 with the identical antigen or hybrids. The mice were bled at weeks 4 for the evaluation of F306-specific serum antibodies by ELISA, in which His-F306 was used as the coating antigen. Each bar represents mean ± SD values obtained from individual mice (5 mice per group). These experiments were performed three times with similar results. The antibody titers between all of two groups had significant differences (P < 0.01).

To establish whether Ii-segments, which bound and co-localized with MHC class II molecules, improved immune response, we immunized mice with these hybrids, including F306 alone, and measured the specific antibody levels. As illustrated in Figure 4, we detected an antibody titer of 1.62 ± 0.53 × 10^4^ using ELISA in mice immunized with F306 alone. The Ii-key/F306 or Ii-key/F306/DN hybrids induced antibody titers approximately 2-fold (3.12 ± 1.15 × 10^4^) or 4-fold (6.30 ± 1.26 × 10^4^) higher, respectively, compared with F306 alone. Moreover, the hybrids containing the cytosolic and transmembrane domains, Cyt/TM/Ii-key/F306 or Cyt/TM/Ii-key/F306/DN, elicited up to approximately 9-fold higher antibody titer (12.40 ± 1.11 × 10^4^or 13.03 ± 1.56 × 10^4^) than F306 alone. However, the Ii/F306 hybrid induced an antibody titer approximately 3-fold (4.52 ± 1.22 × 10^4^) higher compared with F306 alone. These results suggested that, except for the trimerization region, the tested Ii-segments played a role in boosting the immune response in different ways, affecting the non-PBR of MHC class II molecules and immune cells.

## Conclusions

We demonstrated by immunoprecipitation and western blotting that Ii segment cytosolic and transmembrane domains bound to MHC class II molecules, and by confocal microscopy that they co-localized on the plasma membrane with MHC class II molecules in transfected cells. Although the amino acid sequences lying outside the N and C terminal segments of CLIP, Ii-key and DN, neither bound to nor co-localized with MHC class II molecules, the both segments made the epitope in the hybrids concomitantly more stable when associated with a PBR .

Together with the cytosolic and transmembrane domains, these amino acid sequences associate with the non-PBR of MHC class II molecules to form complexes, hybrid/MHC II, which might be better for presenting epitopes to immune cells, improving specific antibody production.

## Discussion

### Ii-key and DN facilitate epitope loading in MHC II PBR

The conformation of the MHC class II molecules plays an important role in peptide association [35-41]. Binding of peptide to MHC class II molecules involves several conformational changes, including a transient “peptide-receptive” conformation [41-43]. The efficient generation of long-lived peptide/class II complexes involves two stages: initial conditioning of MHC class II molecules in an acidic environment, forming a floppy MHC that increases the ability of class II molecules to enter a compact conformation, upon binding to specific peptides [35]; whereas the mature peptide-loaded MHC class II molecules appear as compact heterodimers [44]. Ii-key and DN lie just outside N and C termini of the CLIP region, respectively. Ii-key facilitates epitope loading of MHC class II molecules at the cell surface [26,27]. DN, two relatively conserved residues and hypothetically similar to Ii-key, help CLIP bind to the MHC class II PBR, enabling effective and stable epitope (F306) loading of the PBR. Furthermore, the MHC class II molecules exist in at least two different conformations with respect to their peptide-binding ability; one more receptive to binding than the other [45]. The peptide may configure a class II MHC structure [35,36], resulting in a compact conformation facilitating charging of epitopes [28]. Ii-key and DN possibly enhance the loading stability of Ii-key/F306 or Ii-key/F306/DN with MHC class II PBR, to induce a conformational change in the trough and facilitate effective epitope charging [28] and generation of long-lived peptide/class II complexes [35], affecting T cell secretion of IFN and increased humoral immune response [28]. However, the affinity between Ii-key and DN, and MHC class II molecule is not sufficient to form complexes in cotransfected cells or immunoprecipitation (Figure 2).

### Cytosolic and transmembrane domain binding to non-PBR improves epitope loading in MHC class II PBR

The Ii and the Ii-segment/F306 hybrids bind MHC class II α or β chains as complexes on the plasma membrane (Figures 2 and 3), based on their trimerization region. The Cyt/TM/Ii-key/F306(CLIP)/DN lacks the trimerization region, although it also binds MHC class II molecules to form visible complexes (Figures 2 and 3). The transmembrane domain has a role in the formation of αβ/Ii trimers [17]: along with Ii-key and DN, it is the third factor for binding to MHC class II molecules and maintaining stability of epitope/MHC II complexes. However, this domain also co-localizes to the membrane with MHC class II molecules. The MHC class II molecules require localization on the membrane rafts for signal transduction [46]. A preferential localization of peptide-bound MHC class II molecules on the membrane results in optimal antigen presentation [47-49]. In addition, the cytosolic domain contains an endosome-targeting signal for immune regulation [15,16]. The binding of the non-PBR via Ii functional segments, co-localization with MHC II on the membrane, and signal transduction are sufficient for stable complex formation, antigen presentation and initial immune response.

### Ii-segments are a potential immune carrier

The Ii/F306 hybrid bound MHC class II molecules strongly to form complexes on the plasma membrane (Figure 3). It stimulated an intermediate immune response, which was lower than that of other hybrids containing cytosolic and transmembrane domains (Figure 3). Under normal conditions, the MHCII/Ii complex is directed to endosomes [50,51] and then to immature lysosomes, where the Ii is proteolyzed [12], and the CLIP is replaced by an antigenic peptide [13]. The Ii/F306 hybrid contains a trimerization region at its C terminus, which enables the Ii hybrid/MHC II molecule to form stable complexes. However, tight binding between MHC class II molecules and Ii/F306 hybrid might prevent Ii/F306 hybrid release to bind other MHC class II molecules and activate other immune cells. In other words, the trimerization region disrupts contact with receptors at other cell surfaces, which is necessary for initiation of the immune response when MHC class II molecules present antigenic peptide. In contrast to Ii/F306 hybrid, the other Ii-segment/epitope hybrids such as Cyt/TM/Ii-key/F306/DN bind MHC class II molecules on the plasma membrane weakly, which is sufficient to form relatively stable complexes to induce an immune response, but also for its disassembly to bind and activate more immune cells. In brief, these Ii-segments may be used as a carrier to promote specific immune responses.

## Methods

### Cloning and construction of the hybrids

We cloned various Ii functional segments from mouse Ii cDNA using PCR and constructed the Ii-segment/F306 hybrids (Figure 1) with a series of primers (Table 1). We also cloned mouse *H2-Aa* and *H2-Ab* genes with the primers (Table 1). An Ii/F306 hybrid, in which the CLIP region was replaced by F306, was constructed by overlap extension PCR. The constructed Ii-segments or hybrids were then inserted into eukaryotic vector pmCherry-C1 or pEGFP-C1 (Table 1, Nos. 4–12), and the mouse *H2-Aa* and *H2-Ab* genes were inserted into pEGFP-N1 (Table 1, Nos. 1 and 2) to enable identification by confocal microscopy. These Ii-segment/F306 hybrids were also inserted into prokaryotic expression vectors pGEX-4 T-1 (Table 1, Nos. 13–18) for immunization antigen. Additionally, F306 was inserted into pET-32a (No. 17) for expression of the coating antigen used in the ELISA. Mouse *H2-Ab* genes were inserted into PCMV-Myc (Table 1, No. 3) for the expression of eukaryotic protein by immunoprecipitation and western blotting. All the constructed hybrids were identified by sequencing.

### Cell culture, transfection and confocal microscopy

COS7 cells were obtained from Biology Science College, University of Science and Technology of China. The cells were grown at 37 °C in the presence of 5% CO_2_ in Dulbecco’s modified Eagle’s medium with 10% fetal calf serum (FCS) (GIBCO, USA). Transfection of COS7 cells was done using Lipofectamine 2000 (Invitrogen) reagent following the manufacturer’s instructions. Briefly, the hybrid vectors and media (amount depending on the size of the well) were mixed together, and Lipofectamine 2000 (twice the amount of the hybrid vector) was mixed with the medium in a separate tube. The medium containing the hybrid vectors and the medium containing Lipofectamine were mixed together, allowed to sit for 15–20 min at room temperature, and added slowly to the well. After 5 h, the medium was replaced with fresh media containing penicillin and streptomycin, and 10% fetal bovine serum. After 24 h, images of the COS7 cells were acquired with a Zeiss confocal laser scanning microscope (CLSM) using a × 60 oil objective [excitation at 488 nm for red fluorescent protein and emission at 515 nm for green fluorescent protein (GFP)].

### Immunoprecipitation and western blotting

Immunoprecipitation included cotransfection of the COS7 cells seeded in 25-cm^2^ plates with fusion genes *H2-Ab*/*Myc* and Ii/GFP, Ii/F306/GFP, Ii-key/F306/DN/GFP, Cyt/TM/Ii-key/F306/DN/GFP or Cyt/TM/Ii-key/F306/GFP, respectively. At 36 h post-transfection, the cells were harvested and lysed in 1 mL immunoprecipitation lysis buffer (50 mM Tris–HCl, 150 mM NaCl, 1% Nonidet P40, and 0.5% sodium deoxycholate, 1 Protease Inhibitor Cocktail Tablets) at 4°C for 1 h. The cells were then centrifuged at 12 000 *g* at 4°C for 1 h, and 20 μL Protein A/G Plus-Agarose beads (GE Healthcare, USA) were added to the supernatants and incubated at 4°C for 2 h. After centrifugation at 12 000 *g* for 20 s at 4°C, 2 μL antibody to Myc (Zhongshan Golden Bridge Biotechnology, Beijing, China) was added to the supernatants and incubated for 2 h at 4 °C. The immune complexes were isolated using 50 μL Protein A/G Plus-Agarose beads at 4°C overnight. Centrifugation involved suspending residue in 1 mL immunoprecipitation lysis buffer, buffer 2 (50 mM Tris–HCl, 500 mM NaCl, 0.1% Nonidet P40, 0.05% sodium deoxycholate) and buffer 3 (50 mM Tris–HCl, 0.1% Nonidet P40, 0.05% sodium deoxycholate) for 20 min and adding Protein A/G Plus-Agarose beads under the above conditions, and repeating three times. Subsequently, washed immunoprecipitates were separated by sodium dodecyl sulfate polyacrylamide gel electrophoresis (SDS-PAGE) and transferred onto the polyvinylidene fluoride membrane (Millipore, Schwalbach, Germany). The blots were blocked with 10% (v/v) FCS for 1 h and then probed for 1 h with a murine antibody to GFP (Zhongshan Golden Bridge Biotechnology), followed by washing and incubation for 2 h with horseradish peroxidase (HRP)-conjugated secondary Abs (goat anti-mouse IgG, Zhongshan Golden Bridge Biotechnology) and an ECL detection system (Pierce Roclford).

### Expression and purification of antigens

A homologous series of F306 or Ii-segments/F306-epitope hybrids cloned into pGEX-4 T-1 and pET-32a was transfected into *E. coli* expression strain Rosetta. Antigen expression was induced by 1 mmol/L IPTG. All proteins were extracted in denaturing conditions according to the Qiagen protocol and purified by immobilized-metal affinity chromatography with Ni-NTA agarose beads following the manufacturer’s instructions (Amersham Biosciences, Little Chalfont, UK), and found to be >98% pure by analytical HPLC. They were dissolved in sterile distilled water (5 mg/mL) and stored at −70 °C.

### Mice and immunization

Balb/c female mice (10 weeks old) were obtained from the Animal Centre of Anhui Medicine University and bred under specific pathogen-free conditions at the facility. All experimental procedures were performed following the Anhui Medicine University animal care guidelines under an approved protocol. Thirty five mice were divided into seven groups. Mice to be immunized were anesthetized and injected intraperitoneally with 50 μg of each protein antigen. The animals received the protein doses at week 0, with complete Freund’s adjuvant as a 1:1 (v/v) emulsion in 100 μL. The second immunization occurred at weeks 2 in incomplete Freund’s adjuvant, and the third immunization took place at week 3 without adjuvant were carried out. One control group of mice was injected as above without any antigen. The sera were prepared at week 4 from blood collected from mice via the tail vein, and were stored at −20°C until used for estimation of the antibody titers.

### Detection of antibody with ELISA

Ninety-six-well EIA/RIA plates (COSTAR, USA) were coated with 5 μg/mL His-F306 peptide and then blocked with 0.05% Tween-20 in PBS (PBST) containing 1% bovine serum albumin. The sera were added to the top row of each plate, and serial 1:2 dilutions in PBST were then placed in subsequent rows. The plates were incubated for 45 min at room temperature and washed with PBST. A goat anti-mouse IgG HRP conjugate (Zhongshan Golden Bridge Biotechnology) diluted 1:5000 was used as a secondary antibody and incubated for 45 min, followed by addition of OPD peroxidase (Sigma, USA) used as a substrate. After 15 min of incubation at room temperature, the absorbance was measured at 405 nm.

### Statistical analysis

Statistical differences were calculated by one-way analysis of variance with post-test. Significance was defined as *P* < 0.01. All functional assays, e.g., specific antibody titers in ELISA, were performed in quadruplicate.

## Competing interests

The authors declare that they have no competing interests.

## Authors’ contributions

CF carried out the cloning, construction and identification of the hybrids, analysis of immunoprecipitation and western blotting, and drafted the manuscript. FM performed expression and purification of antigens, immunization of mice, detection of antibody with ELISA, and statistical analysis. LP performed the cell culture, transfection, and confocal microscopy. FX assisted with expression and purification of antigens. XL assisted with detection of antibody with ELISA, and statistical analysis. All authors read and approved the final manuscript.
